# The management of childhood urinary incontinence

**DOI:** 10.1007/s00467-014-2791-x

**Published:** 2014-03-11

**Authors:** Michal Maternik, Katarzyna Krzeminska, Aleksandra Zurowska

**Affiliations:** 1 Department of Pediatrics, Nephrology, Hypertension, Medical University of Gdansk, Gdansk, Poland; 2 Department of Physical Therapy, Medical University of Gdansk, Gdansk, Poland

**Keywords:** Children, Incontinence, Enuresis, Dysfunctional voiding, Overactive bladder, Lower urinary tract

## Abstract

The International Children’s Continence Society (ICCS) has undertaken an enormous effort to standardize both the terminology and management of various aspects of incontinence in children, including enuresis, bladder overactivity, dysfunctional voiding and psychological comorbidities. A number of guidelines have been published to aid those involved in the care of children with lower urinary tract symptoms. This review addresses a number of recommended diagnostic and therapeutic strategies, including urotherapy and pharmacological treatment, with emphasis on a focused medical history, information acquired from bladder diaries and uroflow evaluations. The major role of urotherapy is underlined with supportive pharmacotherapy, when indicated. The article provides both a summary of ICCS guidelines and a brief review of recently published papers related to the contemporary management of childhood incontinence, a health issue still underestimated by both the child’s caregivers and healthcare providers.

## Introduction

### Overview of incontinence

An immense effort has been made by the International Children’s Continence Society (ICCS) to standardize the terminology of lower urinary tract (LUT) dysfunction in order to avoid misunderstanding among all of the parties taking care of children with LUT symptoms [1]. Detailed guidelines for the diagnosis and treatment of the basic problems associated with incontinence, as well as a list of clinically useful materials (frequency/volume charts, bowel diaries) for the management of children with this distressing condition, may be found on their website (www.i-c-c-s.org).

According to the ICCS standardization document incontinence may be intermittent (more common) or continuous (implying an anatomical and/or neurological deficit). Intermittent incontinence is applicable to children who are at least 5 years of age, and it is further classified as either night (enuresis) or day wetting and as either primary or secondary (occurring after a period of dryness of at least 6 months) [1]. The incidence of incontinence in childhood is high. A recent study carried out in the UK reported a 15.5 % rate for enuresis in 7.5-year-old children, which decreased with age but remained at 0.5–1 % in adults [2]. Daytime incontinence has been reported in 15 and 5 % of 4.5- and 9.5-year-old children, respectively [3]. This distressing symptom is present in a variety of underlying conditions, and the correct recognition of the condition will determine the appropriate choice of treatment. Due to the close anatomical and functional relationship between the lower gastro-intestinal tract and the bladder, an adequate assessment of both is crucial. Constipation needs to be diagnosed and treated before and throughout incontinence therapy [4]. The rate of comorbid behavioral and emotional disorders in children with incontinence is high: epidemiological studies have shown that 20–30 % of children with nocturnal enuresis, 20–40 % with daytime urinary incontinence and 30–50 % with fecal incontinence meet the criteria for psychiatric disorders. Consequently, physicians should have a basic understanding of psychological principles and apply the same care in the assessment of behavioral aspects as is used to exclude the less frequently present organic causes of incontinence [5].

### Evaluation

History-taking is invaluable and although it is time-consuming, the results of a well-focused medical history may directly lead to a correct diagnosis without the need for additional tests and examinations [1, 6, 7]. Based on medical history, patients can be classified into the following basic subgroups: monosymptomatic nocturnal enuresis (MNE), in whom only nocturnal enuresis is present and non-monosymptomatic nocturnal enuresis (NMNE), in whom nocturnal enuresis is accompanied by other LUT symptoms. This classification is clinically relevant as these subgroups differ in terms of pathophysiology and available therapy options. As such, it is important to document all LUT symptoms, such as an increased/decreased voiding frequency, daytime incontinence, urgency, hesitancy, straining, a weak stream, intermittency, holding maneuvers, a feeling of incomplete emptying, post-micturition dribble and genital or LUT pain [7]. A full medical history should also include drinking habits, bowel symptoms, any previous interventions and psychological aspects.

The second step in the structured diagnostic evaluation of a child with incontinence is the physical examination. Special attention should be paid to abdominal palpation in order to exclude the presence of organomegaly, bladder distension or fecal masses and to inspection of the genital and sacral regions to exclude congenital malformations and cutaneous manifestations of spinal dysraphism [6].

Frequency/volume charts and bladder diaries are tools without which the reliable management of incontinence is impossible. The information derived from these simple and easily available charts is not only of diagnostic, but also of therapeutic value for the child and their family. They will allow the child’s caregivers to document maximal and average voided volumes, voiding frequency, incontinence episodes, bowel movements, fluid intake and urine production [1].

Behavioral comorbidities always need to be taken into consideration. These can be screened for using a recently published short, standardized questionnaire that has been specifically designed for children with enuresis [8]. If behavioral comorbidities are recognized, further counseling is recommended [5].

Further additional examinations to be considered include urinalysis, ultrasonography and uroflow measurement. Urinalysis will exclude urinary tract infection (UTI) as a possible cause of the existing LUT symptoms. Ultrasonography may be useful to recognize kidney and urinary tract abnormalities and evaluate the bladder before and after micturition. It also enables assessment of rectal distension caused by constipation [9]. There is, however, scant evidence for the standard use of ultrasound in every child with MNE. Non-invasive uroflow with pelvic floor electromyography is helpful in identifying dysfunctional voiding [1]. Further invasive procedures, such as voiding cystourethrogram (VCUG), urodynamic investigation or spinal cord magnetic resonance imaging (MRI), should be reserved for patients with suspected organic uropathies and neurological problems, or those not responding to first-line treatment [6].

### Treatment

The basic therapeutic strategy for children with incontinence is urotherapy. Urotherapy refers to nonpharmacological and nonsurgical interventions used to treat LUT and bowel dysfunction. Standard urotherapy includes education on normal LUT function, regular voiding habits and voiding posture, life-style advice regarding fluid intake and prevention of constipation and instruction on the use of bladder diaries or frequency–volume charts. Urotherapeutic interventions encompass various methods of pelvic floor muscle (PFM) training, behavioral modification, neuromodulation and catheterization.

Behavioral problems, if identified, need to be treated as they affect both compliance and motivation of incontinence therapy. Pharmacological treatment may or may not be added to urotherapy as indicated.

## Enuresis

Enuresis is the most common pathology related to the urinary tract in children. According to ICCS recommendations enuresis is defined as monosymptomatic (enuresis without any other LUT symptoms present during the day) or nonmonosymptomatic (if accompanied by daytime symptoms) [1]. Two main pathophysiological causes of enuresis are recognized: nocturnal polyuria, where decreased nocturnal vasopressin levels have been demonstrated, or decreased bladder storage during the night, which is frequently associated with overactivity of the bladder (OAB) [10, 11]. In both situations sleep disturbances may play an important role. Although an increased arousal threshold in children who do not wake to impulses from the bladder is the most commonly implicated mechanism, more complex pathways involving the sleep–awake cycle are being recognized and evaluated [12–15].

### Treatment

The general approach to the management of intermittent nocturnal incontinence is initial treatment of accompanying daytime symptoms or of constipation, if present. Specific enuresis interventions include urotherapy, alarm treatment and pharmacotherapy.

General lifestyle advice is given as part of standard urotherapy. The child and their family should be informed about the function of the urinary tract and the causes of enuresis and thereafter be given advice on appropriate fluid intake, regular voiding during the daytime and emptying the bladder before going to bed. Special attention should be paid to drinking habits. Regular fluid intake during the day and minimization of fluid and solute intake 1–2 h before sleep is encouraged in order to reduce increased nocturnal urine production. A calendar of dry and wet nights should be kept for self-monitoring and motivation. Alarm treatment or desmopressin therapy should be discussed with the patient and their family. Both methods are equivalent first-line treatment options which can be proposed by the general practitioner. The choice of initial treatment may be based on the parents’ and child’s preference, the physician’s experience and local resources. A proposed optimal treatment strategy is the introduction of desmopressin in the presence of nocturnal polyuria [urine production exceeding 130 % of the expected bladder capacity (EBC) for a given age] and the choice of the alarm for children with decreased maximal voided volumes. Combined therapy with desmopressin and the alarm may be effective in those children in whom both mechanisms are at work or overlap [7, 16].

#### The alarm

Alarm therapy is based on the use of electronic devices which sense moisture and trigger an alarm connected to the device. The sensor can be placed in the child’s underwear or pad near the perineum (a personal alarm) or in a mat on which the child lies (a pad and bell alarm). Most alarms generate an acoustic signal, although alarms with vibrations and light effects are also available. Alarm therapy is curative in 60 % of children through conditioning effects on arousal and/or increasing bladder volume [17]. The minimal duration of therapy is 3 months, and it may be stopped after the child has achieved at least 14 consecutive dry nights. Motivation and support provided to the parents and child is crucial: the child needs to learn to respond to the alarm (i.e. wake up), void in the toilet and re-attach the alarm for the rest of the night. Recurrence of enuresis may be observed, but re-introduction of this therapy is often successful [7, 16].

#### Desmopressin

Desmopressin is an antidiuretic vasopressin analog which leads to a decrease in urine production during the night. Approximately 70 % of patients respond to desmopressin, but a high recurrence rate is observed following cessation of this therapy [7, 16]. The regular dosage is 0.2 mg of tablet formulation, equivalent to 0.12 mg of melt tablets. The medication should be taken at least 1 h before sleep to reach a maximal effect, and fluid intake needs to be stopped 1 h before administration. If complete response is not observed, the dosage may be increased to 0.4 or 0.24 mg, respectively. Patients with night-time polyuria and no bladder overactivity are the best responders [18]. The advantage of this treatment option is an immediate response, and it is therefore useful for less motivated families. The treatment duration is 3–6 months, with 1-month breaks to check if enuresis has resolved. Desmopressin is a safe drug even with extended use, but the caregivers and the child need to be warned that excessive fluid intake just before or after drug administration may cause water intoxication, hyponatremia and convulsions [7, 16].

#### Anticholinergic drugs

Although anticholinergic drugs are not the first-line treatment option for nocturnal enuresis, they are used in combination with other treatment modalities for resistant cases [19]. Several studies have shown a significant improvement after the addition of oxybutynin to desmopressin therapy [20]. Standard dosages of 5 mg of oxybutynin or 0.4 mg/kg propiverine at bedtime have been used [20]. Suppression of the overactive detrusor muscle may be beneficial in children with decreased voided volumes and enuresis. Before antimuscarinic drugs are introduced, constipation, post-void residual (PVR), low voiding frequency and dysfunctional voiding need to be excluded.

#### Centrally active medications

Several placebo controlled studies have demonstrated the efficacy of imipramine, a tricyclic antidepressant, for the treatment of nocturnal enuresis [21, 22]. Although the mechanism by which this is achieved is still unclear, it has been postulated that this drug has antidiuretic properties and also lowers the arousal threshold [23]. Imipramine is not available in some countries due to its potential cardiotoxic effects. Screening for long QT syndrome before starting treatment has been proposed, although a normal electrocardiogram does not exclude the possibility of developing arrhythmias. The initial dosage of imipramine in children is 25–50 mg to be taken at bedtime. A re-evaluation is performed after 1 month of therapy; if full response has been reached, the drug is tapered to the lowest effective dose. There should be a 2-week break in therapy every third month to allow evaluation of the resolution of enuresis. Imipramine should be used with caution and looked upon as a tertiary treatment option to be prescribed in specialist centers for the treatment of resistant enuresis. Imipramine may be combined with either alarm therapy or desmopressin [16].

## Day wetting

Lower urinary tract symptoms and day wetting occur in children due to a variety of causes which include an OAB, voiding postponement, an underactive urinary bladder (UUB), obstruction and dysfunctional voiding [1]. Other less likely conditions are stress incontinence (occasionally associated with obesity or chronic coughing), vesico- or urethro-vaginal reflux and giggle incontinence.

### Overactive bladder

Urgency is the main symptom of an OAB, which is termed detrusor overactivity when confirmed by urodynamic investigation. It is accompanied by increased voiding frequency, decreased voided volumes and frequently by incontinence. Children with OAB may demonstrate only urgency or urgency with incontinence of small volumes of urine, or involuntary complete bladder emptying. Uninhibited detrusor contractions lead to characteristic behavior patterns aimed at postponing wetting, such as tiptoeing, forceful leg crossing or squatting with the heel pressed into the perineum [1].

Treatment of this condition includes urotherapy with or without pharmacotherapy. Both treatment options are complementary and their combined use seems to be more effective [24]. Urotherapy involves the active management of bowel dysfunction, implementation of age-appropriate regular fluid intake and regular visits to the toilet. Charting facilitates compliance and motivation. A timer watch can remind the child of toilet visits and routine hydration. A personal wetting alarm during the day may be helpful for carefully selected children with concentration problems [24, 25].

#### Pharmacotherapy (oxybutynin, propiverin, tolterodin, solifenacin)

Pharmacotherapy is introduced if the above interventions are not sufficient to achieve continence. Antimuscarinic agents which block acetylcholine receptors will inhibit the overactive detrusor muscle. Unfortunately, most of the drugs currently available are unselective and additionally interfere with the muscarinic receptors localized outside the bladder, leading to unwanted side-effects, of which the most common are dry mouth, constipation and blurred vision, which in turn affects compliance. These side-effects are caused by inhibition of M3 receptors present in the salivary glands, lower bowel and ciliary smooth muscles [25]. Patients may also demonstrate psychological or personality changes, as well as sympathetic system activation with facial flushing, heat stroke, tachycardia, drowsiness or headache. About 10 % of patients are reported to discontinue therapy due to these side-effects [26]. Special attention should be paid to the presence of constipation; this needs to be actively treated before the introduction of antimuscarinic therapy. PVR urine also needs to be assessed before therapy and subsequently monitored throughout therapy, as a residual can develop with time. Treatment lasts 6–12 months, and the dosage should be tapered slowly.

##### Oxybutinin

Oxybutynin has been the most widely used antimuscarinic drug. It is the only anticholinergic approved by the U.S. Federal Drug Administration for use in children with OAB. The recommended maximal dosage is 0.4 mg/kg of body weight. Immediate release (IR), extended release (ER) and transdermal forms of the drug are available. Oxybutinin is an unselective antimuscarinic drug with a high side-effect ratio, particularly for the IR formulation (dry mouth reported in 17–97 % of subjects, constipation in 5–50 % and abnormal vision in 3–24 %); a better tolerance was observed for the ER and transdermal forms [27]. It has been suggested that the side-effects of oxybutynin are fourfold more common in children than in adults [28].

##### Propiverine

Propiverine is an antimuscarinic with additional calcium channel modulating properties which has been accepted for pediatric use in selected European countries. The suggested pediatric dosage is 0.8 mg/kg/24 h. Studies presented by Marschall-Kehrel et al. confirmed the efficacy of this drug compared to placebo [29]. During a multicenter cohort study the efficacy of propiverine and oxybutynin was similar, but the former was associated with significantly fewer side-effects, such as dizziness, nausea and hot flushes [30].

##### Tolterodine

Tolterodine was introduced as a bladder selective agent for OAB. Several studies in the adult population that have compared tolterodine to oxybutynin reported a similar efficacy but that the former had fewer side-effects [31]. The available data in children are conflicting. Initial retrospective studies in children seemed promising, confirming the superiority of tolterodine over oxybutynin in terms of side-effects [32], but subsequent randomized, double-blinded, clinical trials failed to show any statistical difference between tolterodine and placebo, or tolterodine and oxybutynin [33, 34]. Tolterodine has not been approved for use in children to date and therefore has no recommended pediatric dosage. Nevertheless, according to Hjälmås et al., the adult dosage of 2 mg twice daily is comparable to 1 mg twice daily in children aged 5–10 years [35].

##### Solifenacin

Solifenacin is a novel, selective antimusarinic with moderate selectivity for M3 over M2 receptors. It has not been approved for pediatric use, but studies have shown a good response and tolerance of this agent in children previously resistant to oxybutynin or tolterodine [36]. In a retrospective study of 138 children with OAB refractory to other antimuscarinic agents, 5 mg of solifenacin was associated with an 85 % response rate (full and partial) and a 6.5 % dropout due to side-effects [37]. A multicenter prospective clinical trial is currently underway with a liquid suspension of solifenacin, and a pediatric dosage and approval for pediatric use may be established in the near future [38].

#### Botulin toxin

Botulin toxin A (BTA) treatment is a third-line option for children with idiopathic detrusor overactivity which has been refractory to noninvasive procedures such as urotherapy, PFM training, neuromodulation and pharmacotherapy [39].

BTA is a neurotoxin produced by* Clostridium botulinum* which causes bladder smooth muscle relaxation through inhibition of acetylcholine release at the presynaptic cholinergic junction [40]. BTA has been widely used off label in the treatment of children with neurogenic detrusor overactivity. Dosages between 5 and 12 U/kg body weight have been used with good results [39–41]. During cystoscopy multiple (20–50) injections of BTA into the detrusor muscle are made, bypassing the trigone. Several recent publications have reported the use of BTA for idiopathic detrusor overactivity in children refractory to other forms of therapy [41]. McDowell et al. presented a series of 57 patients with OAB resistant to standard urotherapy who were treated with BTA and antimuscarinics [42]. These authors observed success in 74.2 % of males and 54.5 % of females and partial success in 20 % of males and 18.2 % of females [42]. The effect is unfortunately not permanent, lasting only 6–9 months, and repeated injections are necessary. At the present time there are no ongoing studies evaluating the risk of fibrosis and alteration of bladder compliance following BTA therapy in children with idiopathic detrusor overactivity. Further treatment concerns are urinary retention, cystitis and risk of UTI.

#### Neuromodulation

Neuromodulation is based on the principle that neural structures of the central nervous system may be activated through the use of an electrical current which in turn may modulate the innervation of the bladder. Several studies have shown that treating children with OAB using electrical current at a frequency between 10–25 Hz is a successful therapeutic approach. [43–46]. Neuromodulation may be an alternative treatment to pharmacotherapy for OAB due to its efficacy and lack of side-effects [47].

The treatment methods currently available include parasacral transcutaneous electrical nerve stimulation (TENS; parasacral), percutaneous tibial nerve stimulation/Stoller afferent neuro-stimulation (PTNS/SANS) and implanted sacral nerve stimulation (implanted SNS). The most commonly used of these is home-based TENS with transcutaneous electrodes. Percutaneous neuromodulation (PTNS) has also been shown to be an effective method for the treatment of OAB: Hoebeke et al. reported that 80 % of children improved following this form of therapy [47]. Similar results have been reported for parasacral TENS [48]. Nevertheless, further randomized trials of larger patient groups are necessary to establish the role of this method of therapy.

### Voiding postponement

Some children with daytime wetting and urgency habitually postpone micturation and defecation. This is frequently observed during absorbing daytime activities, such as while playing computer games or with contemporaries. The child postpones bladder emptying as long as possible and consequently wets his/her underwear due to an uninhibited reflex from an overfilled bladder. This postponement may be associated with constipation, as the child may also avoid potentially painful defecation. Over time, constipation may lead to fecal incontinence [1, 49]. Children demonstrating these symptoms frequently suffer from psychological disturbances [50].

The main aim of urotherapy is to correct the frequency of micturition, ensure reasonable fluid intake and stop or reverse bladder overdistension. The monitoring of regular voiding and drinking with the aid of frequency/volume charts or bladder diaries and repeated uroflow and post- void residual evaluations are necessary to assess the efficacy of ongoing treatment [49].

### Underactive urinary bladder

Children with decreased voiding frequency and increased bladder volume exceeding 150 % expected bladder capacity usually have UUB. This condition is related to a decreased ability of the detrusor muscle to contract. The presence of an UUB is indicated by an intermittent flow pattern and can be confirmed by an urodynamic investigation. Children may demonstrate infrequent micturition, an interrupted flow pattern and the need to strain during voiding. An UUB may be caused by neurologic (myelomeningocele, sacral agenesis, cerebral palsy), anatomic (posterior urethral valve, bladder outlet obstruction), functional (voiding postponement, dysfunctional voiding, constipation) or metabolic disorders (mitochondrial diseases) [1, 49].

Muscarinic receptor agonists (bethanechol or carbachol) or inhibitors of acetylcholine esterase (distigmine, pyridostigmine or neostigmine) have been used in the therapy for UUB. Recent studies do not support the previous use of parasympathomimetics for treating UUB [51].

The aim of urotherapy in the treatment of the underactive bladder is to optimize bladder emptying and improve bladder sensation. Education of the child and family consists of introducing regular voiding and drinking regimes, correcting voiding posture and PFM relaxation. Double voiding is a useful technique when increased PVR are observed.

Clean intermittent catheterization may be necessary if UTI, incontinence and incomplete emptying do not respond to urotherapy. A standard regimen consists of five catheterizations daily (every 2–3 h). Hydrophilic-coated catheters seem to be a better choice than uncoated ones, particularly in boys in whom a painful insertion may cause treatment refusal [52]. In children with UUB, attention should be paid to overnight bladder function in order to prevent further overdistension [49]. An overnight catheter may be helpful when UUB coexists with polyuria (early stages of renal insufficiency or diabetes insipidus).

### Dysfunctional voiding

Dysfunctional voiding (DV) is defined and characterized by a staccato uroflow pattern. This condition occurs due to incomplete relaxation of the PFM and sphincters during bladder emptying, a mechanism that is also implicated in the incomplete emptying of the bowel which is frequently associated with DV. DV can co-exist with bladder overactivity, wetting and UTI. In response to a persisting functional obstruction to flow, DV may lead to decompensation of the detrusor muscle and bladder underactivity with resulting overflow incontinence [1]. It may be helpful to discriminate whether the internal or external sphincter is the cause of outflow obstruction. The external urethral sphincter is involved in the majority of children, and for these children, PFM retraining is the first-line treatment. The treatment for primary bladder neck dysfunction, when the internal sphincter does not relax, is different [53].

#### Treatment

##### Urotherapy

Urotherapy and muscle retraining will be successful for the majority of patients with dysfunctional voiding. The aim of urotherapy is to normalize bladder emptying and storage by teaching relaxed voiding techniques. Fluid and voiding regimes are also an essential part of the treatment. The first step in achieving bladder emptying with a relaxed pelvic floor is teaching the correct position and posture during micturition. To urinate correctly, the child needs to have a straight back with the pelvis in a proper position. The feet need to be supported, hips abducted, and buttocks well-supported. Trousers and underpants should be pulled well down below the knees, so they do not obstruct urine flow or limit voiding position [49, 53–55].

Most children will learn relaxed voiding and bowel emptying by being provided with simple instructions from an urotherapist or nurse trained in muscle re-education. Some children will require additional input. Biofeedback is a helpful tool. This method has been used for treating a variety of acquired behavior dysfunctions through the visualization of physiological processes. Treatment of bladder disturbances utilizes EMG tracing of PFM contraction and relaxation visualized in the form of an animation tailored for children, or a simple uroflow curve [56, 57]. Bower et al. reported a success rate of 90 % for children with DV who followed a half-day urotherapy program which included uroflow biofeedback and instruction on the relaxation of PFM [58]. Animated biofeedback programs which involve computer games are particularly suitable for children and enable faster resolution of symptoms when compared to non-animated methods [59]. Several studies have demonstrated a significant improvement in treatment results with the addition of biofeedback to standard urotherapy, but widespread use of this method is hampered by the requirement of a dedicated staff and availability of equipment [60].

##### Pharmacotherapy

According to the ICCS statement there is no approved pharmacological treatment for dysfunctional voiding, but it may be considered when other forms of treatment have failed [49]. The data on the use of alpha-blockers are conflicting. Improvement in Qmax or PVR has been reported, but therapy has usually been combined with biofeedback [61, 62]. Preliminary data have been published on the use of tizanidine, a muscle relaxant, but more studies are needed to assess its efficacy [63]. In primary bladder neck obstruction, the alpha-receptors in the bladder neck have been a target for pharmacological treatment. Alpha-blockers have been used in order to decrease bladder neck resistance and improve bladder emptying [54, 64, 65].

Promising data on the efficacy of botox injections into the external sphincter have been reported in a small group of children with DV resistant to other therapies [66, 67]. BTA leads to temporary paralysis of the external sphincter and may decrease functional bladder outlet obstruction. It has to be stressed that this is an experimental treatment which may be considered only if other forms of therapy fail.

### Stress incontinence

Stress urinary incontinence (SUI) is the involuntary leakage on effort, exertion, coughing or sneezing and is relatively rare in neurologically intact children [1, 68, 69]. It may be observed in adolescent girls with chronic cough, such as that associated with cystic fibrosis or obesity. In children, wetting during increased intra-abdominal pressure is more likely to be due to masked OAB or an over-distended bladder. Stress incontinence is recognized by an urodynamic investigation. Involuntary leakage is observed during increasing intra-abdominal pressure due to inadequate urethral closure pressure.

Children with SUI should be advised about regular drinking and voiding and in particular correct bowel habits. In adults, straining during defecation is highly associated with this condition. While there is evidence for the efficacy of PFM training for SUI in adults and young athletes in whom increasing PFM strength has led to improvement of urinary incontinence, reports on the treatment of children are lacking [70].

### Vesico-vaginal reflux

Vesico-vaginal (or urethro-vaginal) reflux is a condition which typically occurs in prepubertal girls. The urinary stream is directed towards the vagina because of the position of the urethral opening, entrapment of urine by the labia or poor toilet posture and compression by the thighs. Wetting occurs later, frequently during physical activities when urine, entrapped in the vagina, leaks out. The hallmark of this condition is post-void wetting. Vesico-vaginal reflux is a possible cause of urine sample contamination and a possible risk factor for UTI [71]. Girls suffering from vaginal reflux should be instructed on how to spread the labia, abduct the thighs and direct the urine stream forward by a non-flexed lumbar spine during voiding. One option is to sit on the toilet backwards because this position facilitates opening the labia and pelvic posture [72]. Treatment of labial adhesions, meatal anomalies and attention to diet (if overweight) may also be helpful [73].

### Giggle incontinence

Giggle incontinence is a rare but disturbing condition. The pathogenesis is unclear, but it may be centrally mediated and potentially is a hereditary disorder. It is characterized by involuntary, complete bladder emptying during laughing with otherwise normal bladder and bowel function [1]. It should not be confused with dampness on laughing, which is a manifestation of OAB.

Rapid and forceful recruitment of the PFM (known in adult literature as “bracing”) can be helpful. Biofeedback has been proposed as an alternative option [74]. Several studies have reported the successful use of methylophenidate, a treatment based on the postulated central system involvement of this condition, but prolonged use of the drug may be necessary [75, 76]. Dosages vary between 0.2 and 0.5 mg/kg daily with an 8-h acting formulation and are administrated just before school time, when the possibility of incontinence episodes is highest [77].

### Extraordinary daytime frequency

Children who suffer from extraordinary daytime frequency syndrome present with an isolated symptom of sudden frequent voiding of small volumes during the daytime without any accompanying nocturia or wetting. Children may void at least once hourly, and average voided volumes are <50 % of EBC [1]. The condition is usually self-limiting. Age-appropriate hydration with noncaffeine or nonacidic fluids is a reasonable approach. Anticholinergic and anti-inflammatory medications have been tried with varying success. Explanation, re-assurance and psychological counseling are undertaken, if required.

## Summary

Urinary incontinence is a common condition in children and may be associated with constipation and psychological or psychiatric disorders and less frequently with structural abnormalities of the urinary tract. The initial diagnostic approach consists of a focused medical history and physical examination, a bladder diary or volume/frequency chart, urinalysis, ultrasound of the urinary tract and screening for the presence of behavioral problems. Further investigations will be necessary in some children and may include uroflow and urodynamic investigations, VCUG or MRI of the spinal cord. Urotherapy is a first-line treatment for most conditions related to incontinence, but pharmacological treatment may also be helpful. Most children with enuresis, the most common condition related to incontinence, can be treated by a primary care physician or pediatrician. Other conditions may require the expertise of a multidisciplinary pediatric team (nephrologist, urologist, nurse, physiotherapist, gastroenterologist, psychologist, psychiatrist) for appropriate management. Although urotherapy is a first-line treatment for most LUT dysfunctions, standardization of this method is lacking. Further studies are necessary to compare the efficacy of various urotherapeutic interventions. Likewise, there is a need for pediatric clinical trials of the newer pharmacological agents.

## Questions (answers are provided following the reference lsit)

1. Children with urinary incontinence have high comorbidity with:
  - Congenital malformations of urinary tract
  - Behavioral and psychiatric disorders
  - Obesity
  - Hypertension
2. The first line pharmacological treatment for enuresis is:
  - Oxybutynin
  - Imipramine
  - Desmopressin
  - Solifenacin
  - Botulin toxin A (BTA)
3. Urotherapy includes:
  - General lifestyle advice about proper fluid intake and diet
  - Pelvic floor muscle retraining
  - Neuromodulation
  - All of the above
4. The second-line treatment for children with an underactive bladder, significant post void residual and UTI who do not respond to urotherapy is:
  - Oxybutynin
  - Clean intermittent catheterization
  - Desmopressin
  - Parasympathomimetics
5. Detailed guidelines on the diagnosis and treatment of LUT dysfunction have been published by:
  - ICCS (International Children’s Continence Society)
  - ESPU (European Society for Pediatric Urology)
  - ESPN (European Society for Pediatric Nephrology)
  - AUA (American Urological Association)

## Abbreviations

DV: Dysfunctional voiding
ICCS: International Children’s’ Continence Society
LUT: Lower urinary tract
PFM: Pelvic floor muscles
UTI: Urinary tract infection
